# Machine Learning to Enhance Biophysical Models for Riboswitch Discovery

**DOI:** 10.1002/wrna.70062

**Published:** 2026-10-05

**Authors:** Rami Zakh, Alexander Churkin, Ahmed Elbanna, Sumit Mukherjee, Danny Barash

**Affiliations:** ^1^ Institute for Interdisciplinary Computational Science Ben‐Gurion University Be'er‐Sheva Israel; ^2^ Department of Software Engineering Sami Shamoon College of Engineering Be'er‐Sheva Israel; ^3^ Mathematics Department, Faculty of Science Tanta University Tanta Egypt; ^4^ Cancer Data Science Laboratory, Center for Cancer Research National Cancer Institute, National Institutes of Health Bethesda Maryland USA

## Abstract

Riboswitches are RNA‐based genetic control elements that provide a unique mechanism of gene regulation. They function without the participation of proteins and are believed to represent ancient regulatory systems on the evolutionary timescale. To expand the search for novel riboswitches of significant interest, such as eukaryotic riboswitches beyond the purine riboswitch class, the integration of machine learning into RNA design offers considerable potential to improve the effectiveness of traditional search methods. Many riboswitch aptamers that could be targeted in such searches are larger than purine riboswitch aptamers, making their design time‐consuming (e.g., SAM, lysine, FMN, glycine, and cobalamin riboswitches). In an example problem unrelated to the discovery of novel riboswitches, namely challenges in the eteRNA game, it has been shown that a transformer encoder‐decoder model is effective in solving difficult inverse RNA folding challenges. In the outlined approach, machine learning is used as a preprocessing step before RNA design, rather than relying on random inputs to the Monte Carlo method, and the RNA design step crucially depends on a biophysical model for the forward problem of RNA folding prediction. It is therefore suggested to optimize the training of the machine learning model and then utilize this approach for riboswitch discovery to make searches for riboswitches larger than purine aptamers more efficient and robust.

## Graphical abstract

An encoder–decoder transformer–based machine learning model is used to enhance inverse RNA folding. It operates as a preprocessing step for a solver that employs a biophysical model to predict RNA secondary structure from a given sequence.

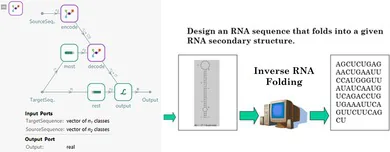

## 1 Introduction

Genetic control of fundamental processes such as transcription, translation, and splicing is known to be a complex process. A significant number of proteins monitor the environment and selectively bind to target genes to regulate their expression. Surprisingly, RNA genetic control elements were discovered that can sense small ligands and assume a regulatory role by switching states without the participation of proteins. These RNA elements are called riboswitches, as described in early reviews by Mandal and Breaker (2004) and Nudler and Mironov (2004). Although their discovery was biological, with the first experimental validations published in 2002 (Nahvi et al. 2002; Mironov et al. 2002; Winkler, Nahvi, et al. 2002; Winkler, Cohen‐Chalamish, et al. 2002), conserved sequence patterns in the 5′UTRs of bacteria were identified by comparative analysis of upstream regions of several genes expected to be co‐regulated. These works contributed to the description of the S‐box (Grundy and Henkin 1998), the RFN element (Gelfand et al. 1999), and the THI‐box (Miranda‐Ríos et al. 2001).

In transcription termination during the thiamin (vitamin B_1_) biosynthesis in *Bacillus subtilis* and translation initiation in *Escherichia coli* , respectively, it was shown that the RNA element can respond to a change in the concentration of thiamine pyrophosphate (TPP) with a conformational rearrangement. The capability of this RNA element, namely a “TPP‐riboswitch,” provides an RNA control mechanism in prokaryotic transcription termination and translation initiation. Other riboswitches were found including flavin mononucleotide (FMN), *S*‐adenosylmethionine (SAM), coenzyme B_12_, lysine, purine, as well as second messengers such as glucosamine‐6‐phosphate, glutamine, glycine, cyclic di‐GMP, and several others that have been reviewed in Serganov and Nudler (2013) and Kavita and Breaker (2022). The structural basis and biochemical properties of several of these riboswitches have also been investigated at high resolution in their tertiary structure, for example, Kwon and Strobel (2008) for the glycine riboswitch and Shanahan et al. (2011) for the cyclic di‐GMP riboswitch. The structure of the earlier discovered purine riboswitch, which is the subject of this proposal, has been experimentally examined in depth (Batey et al. 2004; Lescoute and Westhof 2005; Batey 2012). Intriguingly, in contrast to the abundance of riboswitches in prokaryotes, only a single riboswitch was discovered in eukaryotes (the TPP‐riboswitch that has been found in fungi and plants [Sudarsan et al. 2003]).

The discovery of eukaryotic riboswitches is considerably more challenging than that of bacterial riboswitches for several reasons. Compared with bacterial genomes, eukaryotic genomes are often several orders of magnitude larger, have been sequenced less extensively, tend to have less complete gene annotations, all of which make genetic studies generally more challenging. Several factors contribute to these challenges. Eukaryotic gene regulation relies extensively on proteins and is strongly influenced by chromatin and cellular context. Candidate RNAs are less frequently associated with canonical bacterial 5′UTR contexts, and eukaryotic UTRs and coding regions are longer and more heterogeneous. In addition, sequence conservation is often weak, while introns, alternative splicing, RNA editing, and transcript isoform diversity further complicate annotation. Genomic context is also generally less informative than in bacterial operons. Overall, the scarcity of confirmed eukaryotic riboswitches likely reflects both biological and methodological factors. Eukaryotes rely more extensively on protein‐mediated gene regulation, and the characteristics that made bacterial riboswitches relatively straightforward to discover, including strong sequence conservation, predictable genomic organization, and simpler RNA biology, are largely absent in eukaryotic systems.

With the aim of finding all the missing eukaryotic riboswitches, we developed a unique structure‐based search method for riboswitch detection. Our strategy is based on inverse RNA folding that samples the sequence search space more flexibly than any other riboswitch detection strategy to date and can be combined with detection methods like Infernal (Nawrocki and Eddy 2013). It uses structural guidance with RNA design (Mukherjee et al. 2026) as a preprocessing step to help in the search (Drory‐Retwitzer et al. 2015) and was recently extended in a significant way by the ability to construct riboswitch candidate families (Mukherjee et al. 2023). The method efficiently detects the conserved structural signature of existing riboswitch classes by considering the divergence at the sequence level. Using this method, we searched for purine riboswitches in eukaryotic genomes, as these play an important role in the regulation of purine biosynthesis in bacteria (Mandal et al. 2003; Kim and Breaker 2008), yet none were identified in eukaryotes. In this essay, we argue that this method can also be applied to larger aptamers beyond the purine riboswitch aptamer, which we initially chose for convenience. This can be achieved by employing machine learning (ML) as a preprocessing step before RNA design while preserving the use of biophysical models that are fundamental to computational RNA design. We note that, in this Perspective, the term ‘biophysical models’ refers to thermodynamics‐based RNA secondary‐structure prediction models that estimate minimum‐free‐energy structures and/or structural ensembles using nearest‐neighbor parameters. This term does not encompass atomistic molecular dynamics or tertiary‐structure simulations. It is worth emphasizing that our approach for eukaryotic riboswitch discovery is one plausible approach that merits testing; we put these ideas forward to stimulate competing hypotheses. This remains an open problem, and approaches to discovering the missing eukaryotic riboswitches, such as those advanced by Kavita et al. (2025), could lead to important discoveries in the field. Kavita et al. (2025) searched vertebrate RNAs using covariance models based on bacterial guanidine riboswitch aptamers. Using computational methods based on comparative nucleotide sequence analysis (Yao et al. 2006; Nawrocki and Eddy 2013; Weinberg, Lünse, et al. 2017; Weinberg, Nelson, et al. 2017), they searched eukaryotic genomes for RNA domains corresponding to each of the four known bacterial guanidine riboswitch classes (Nelson et al. 2017; Sherlock et al. 2017; Sherlock and Breaker 2017; Lenkeit et al. 2020; Salvail et al. 2020). They focused on the guanidine‐I and guanidine‐II aptamer classes, identified candidate RNAs associated with calcium signaling and genes involved in neuromuscular function, and provided biochemical validation for representative vertebrate guanidine aptamers. Their discovery of vertebrate guanidine‐I and guanidine‐II riboswitch aptamers associated with numerous genes involved in neuromuscular function suggests that guanidine is a widely used signaling molecule that provides an additional layer of gene regulation relevant to neuromuscular disorders. Their approach is complementary to ours because it begins with known bacterial aptamer classes and the associated genomic context, whereas our approach starts from inverse‐folding‐generated candidate RNA families. It is also worth noting that our computational approach is general in that candidates may occur either in coding regions, a possibility suggested by experiments on synthetic riboswitches (Topp and Gallivan 2008), or in non‐coding regions, as has been observed to date.

## 2 ML in Riboswitch Discovery

From the outset, it should be clarified that, in the context of this title, the term ML refers specifically to modern AI approaches based on neural networks, including deep learning and transformer architectures. Classical statistical ML methods already provide highly effective tools for riboswitch identification, and our broader methodology, described in Mukherjee et al. (2023), incorporates the latest version of Infernal (Nawrocki and Eddy 2013) as a major component of its pipeline. Traditional probabilistic and statistical modeling approaches that may also be classified as ML in a more general sense include Infernal (Nawrocki and Eddy 2013), riboswitch scanner with pHMM (Mukherjee and Sengupta 2016), and related methods. In the remainder of this work, however, the term ML will refer specifically to neural‐network‐based models.

### 2.1 Prokaryotic Riboswitch Discovery

Prokaryotic riboswitch discovery in recent years has progressed steadily (Weinberg et al. 2007; Weinberg et al. 2010; Weinberg, Lünse, et al. 2017; Weinberg, Nelson, et al. 2017; Narunsky et al. 2024). As substantial data have been accumulated, an open question is whether ML approaches can be effectively applied to this problem. The available data are inherently heterogeneous, presenting challenges such as multiple ligand classes, uneven class sizes, redundant closely related sequences, class‐specific conserved motifs, taxonomic imbalance, variable annotation quality, and negative examples that have not been experimentally verified as non‐riboswitches. These characteristics influence train/test splitting, model generalization, and evaluation.

To achieve the threshold in data at which ML approaches can become effective, one may need to generate synthetic data in order to train an ML model. In another context, such an idea was explored by Churkin et al. (2022). Although the use of synthetic data is appealing from a computational standpoint, the extent to which it can advance biological understanding remains uncertain. To elaborate, synthetic examples can be obtained by sampling target structures, generating sequences by inverse folding, mutating or augmenting known aptamer families, or folding random or natural sequences and retaining structure‐labeled pairs. Nonetheless, when labels are derived from structure predictions produced by energy‐minimization‐based folding algorithms, ML models may learn the biases of the folding algorithm rather than the underlying biology. Possible mitigation strategies include combining natural and synthetic data, holding out natural riboswitch families during evaluation, benchmarking against experimentally supported structures, comparing predictions across multiple folding algorithms, and explicitly restricting the use of synthetic data to model pretraining or initialization rather than final biological classification. Nevertheless, these limitations should be considered.

### 2.2 Eukaryotic Riboswitch Discovery

Of all bacterial riboswitch classes experimentally validated over the past two decades, only one has been convincingly shown to function naturally in eukaryotes. TPP riboswitches, the most abundant single class in bacteria, are also relatively common in fungi and plants (Sudarsan et al. 2003; Wachter et al. 2007; Bocobza et al. 2007; Mukherjee et al. 2018). More information on this topic, as well as the challenges in identifying the missing eukaryotic riboswitches, can be found in the review by Kavita and Breaker (2022). Advanced bioinformatics approaches beyond the current state of the art may help uncover them.

Thus, there is significant potential for ML methods to address this challenge, particularly when applied creatively. Several approaches are possible. One recent example is described in Raymond et al. (2025), where the authors trained positive and unlabeled ML classifiers on known riboswitch sequences and applied them to *Homo sapiens* mRNA 5′UTR sequences from the UTRdb database to identify candidate mRNAs with potential riboswitch functionality. In their study, 67,683 riboswitch sequences were obtained from RNAcentral and categorized by ligand type to serve as positive examples, while 48,031 5′UTR sequences were used as unlabeled data. At present, the study is purely computational, without experimental validation of the predicted hits. However, the approach could be extended to *Homo sapiens* mRNA 3′UTR sequences and, when combined with experimental validation, may provide a promising strategy for applying ML in this context.

Another distinct approach is proposed in the next section. We suggest using ML as a preprocessing step in the pipeline presented in Mukherjee et al. (2023), with the aim of improving computational RNA design and thereby enabling the exploration of larger riboswitch aptamers than the purine riboswitch aptamer considered in that work. Experimental validation of the computational predictions for the purine riboswitch has been tried, for example, preliminary in vitro experiments such as in Focht et al. (2023), NMR experiments as in Fürtig et al. (2015) by a hypothesis about ATP‐binding aptamers (Trevino et al. 2011) related to Gao et al. (2021) and Mukherjee et al. (2023), in‐line probing (Regulski and Breaker 2008) that was performed in Mukherjee et al. (2023), and others that are currently ongoing.

## 3 ML for RNA Design Augmentation

Some riboswitch aptamers, with the purine aptamer being the best‐known example, can be predicted reasonably well by thermodynamic energy minimization using software such as mfold/UNAFold (Zuker 2003; Markham and Zuker 2008), RNAfold (Hofacker 2003; Lorenz et al. 2011), and RNAstructure (Reuter and Mathews 2010), using experimentally measured thermodynamic parameters (Mathews et al. 1999). This approach was explored in Barash and Gabdank (2010) both for predicting deleterious mutations (Barash and Churkin 2011; Sabarinathan et al. 2013) (that can be supplemented by covariation mutations [Hou et al. 2025], though it remains robust on its own) and for a structure‐based riboswitch search method employing a moving window and folding‐energy predictions. Later, more pragmatic riboswitch search methods based on energy minimization were proposed (Drory‐Retwitzer et al. 2015; Mukherjee et al. 2023). These approaches employ RNA design strategies that fundamentally rely on RNA folding predictions derived from thermodynamic principles.

The incorporation of ML into RNA design as a preprocessing step was first suggested in Brandenburg et al. (2022) for the identification of intrinsic transcription terminators, and later in Cazenave and Touzani (2025) for improving performance on EteRNA game challenges (Koodli et al. 2019). More recently, even without ML, programs such as DesiRNA (Wirecki et al. 2025) and Montparnasse (Cazenave 2025) achieved the capability to solve all EteRNA challenges. It is therefore plausible that machine‐learning‐based preprocessing could further accelerate solution times, as illustrated for DesiRNA in Figure 1A. In Cazenave and Touzani (2025), the data that was used for training is based on Rfam (Ontiveros‐Palacios et al. 2025) sequences, but the data can be chosen randomly and not necessarily based on natural RNA sequences.

**FIGURE 1:**
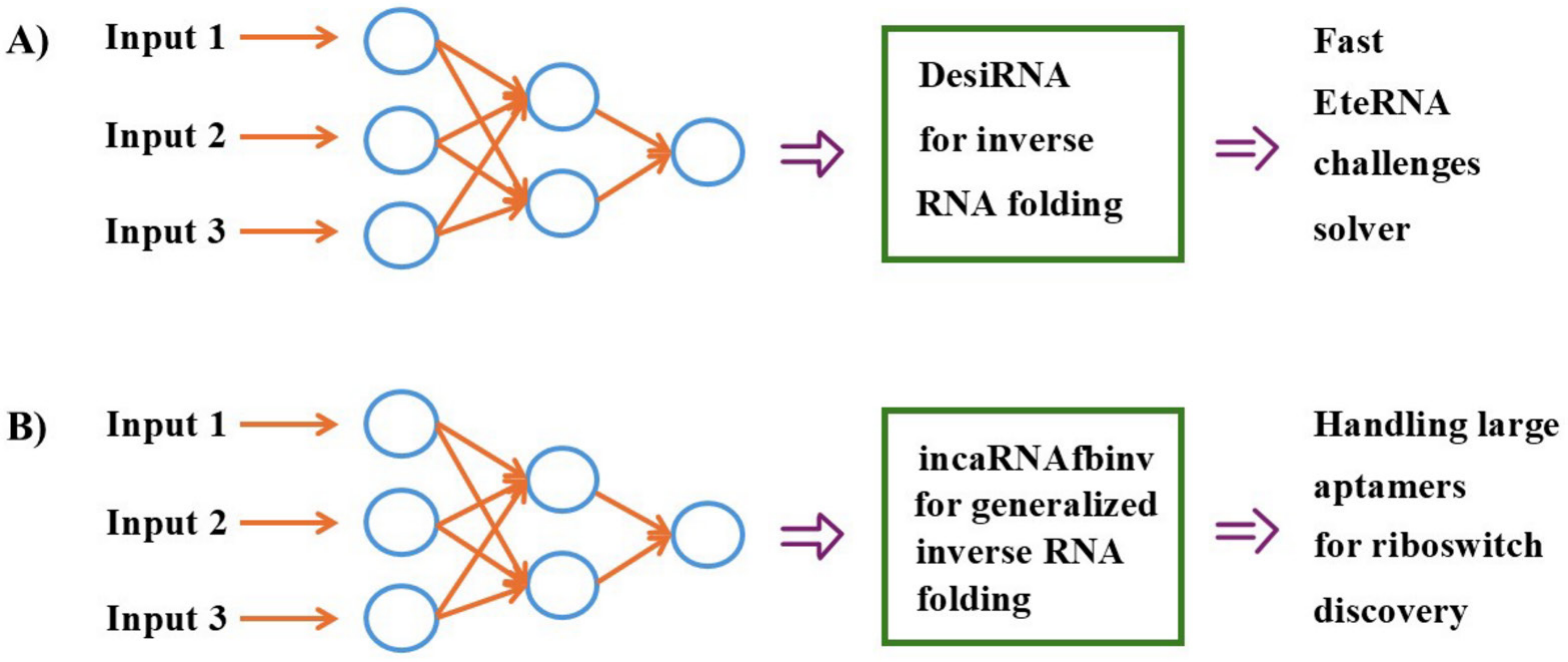
From faster solutions of EteRNA challenges to riboswitch discovery. (A) A generic feedforward network is used to augment DesiRNA (Wirecki et al. 2025) that succeeds to solve all EterRNA challenges in less than 24 h. With machine learning it should be faster. (B) A generic feedforward network is used to augment IncaRNAfbinv (Drory‐Retwitzer et al. 2020) that currently handles the purine riboswitch aptamer for riboswitch discovery. With machine learning, larger riboswitch aptamers should be handled in reasonable time.

DesiRNA employs a replica‐exchange Monte Carlo strategy combined with simulated annealing. Improving the initialization of these optimization runs through Ml could therefore substantially reduce runtime. We argue that a similar strategy could also be extended to riboswitch discovery, as illustrated in Figure 1B, by introducing machine‐learning preprocessing prior to the specialized inverse RNA folding program incaRNAfbinv (Drory‐Retwitzer et al. 2016; Drory‐Retwitzer et al. 2020). Such an approach could extend the riboswitch discovery pipeline outlined in Mukherjee et al. (2023) to larger riboswitch aptamers beyond the purine riboswitch, including the SAM, lysine, FMN, glycine, and cobalamin riboswitches. The rationale is that better initialization enables faster generation of candidate sequences when designing large RNA structures, even though it does not by itself improve homology detection. This is particularly relevant because inverse RNA folding becomes computationally expensive for larger structures, making ML‐guided initialization potentially beneficial.

Based on the runtime comparisons in figure 3 of Drory‐Retwitzer et al. (2016), we estimate that designing a 69‐nt guanine‐binding riboswitch aptamer using incaRNAfbinv with 20 output sequences and 1000 simulated‐annealing iterations takes approximately 2–4 min, whereas designing the substantially longer 127‐nt cobalamin riboswitch aptamer under the same settings takes approximately 9–20 min on dual Xeon E5‐2620 v3 2.4‐GHz processors. It is also worth noting that some structures may be substantially more difficult to design than others. For example, the 40‐nt tripod‐like RNA structure studied by Ivry et al. (2009) took several hours to design using RNAinverse, which has a lower runtime than incaRNAfbinv. In contrast, the guanine‐binding riboswitch aptamer is well predicted by energy‐minimization‐based methods and is known to be relatively easy to design. Thus, these runtime estimates may not be representative of aptamers with more difficult‐to‐design structures, for which ML‐guided initialization could be even more important.

In Cazenave and Touzani (2025) and in a project available on the Wolfram Community (in Further Information), an encoder–decoder Transformer neural network was used for the preprocessing stage. As illustrated in Figure 2, the encoder vocabulary for RNA consists of dot‐bracket secondary‐structure symbols, whereas the decoder vocabulary consists of RNA nucleotides (C, G, A, and U) and base‐pairs (e.g., CG, AU, GU). This architecture, often referred to as a vanilla Transformer model, appears well suited for RNA design augmentation, although alternative architectures may also prove advantageous.

**FIGURE 2:**
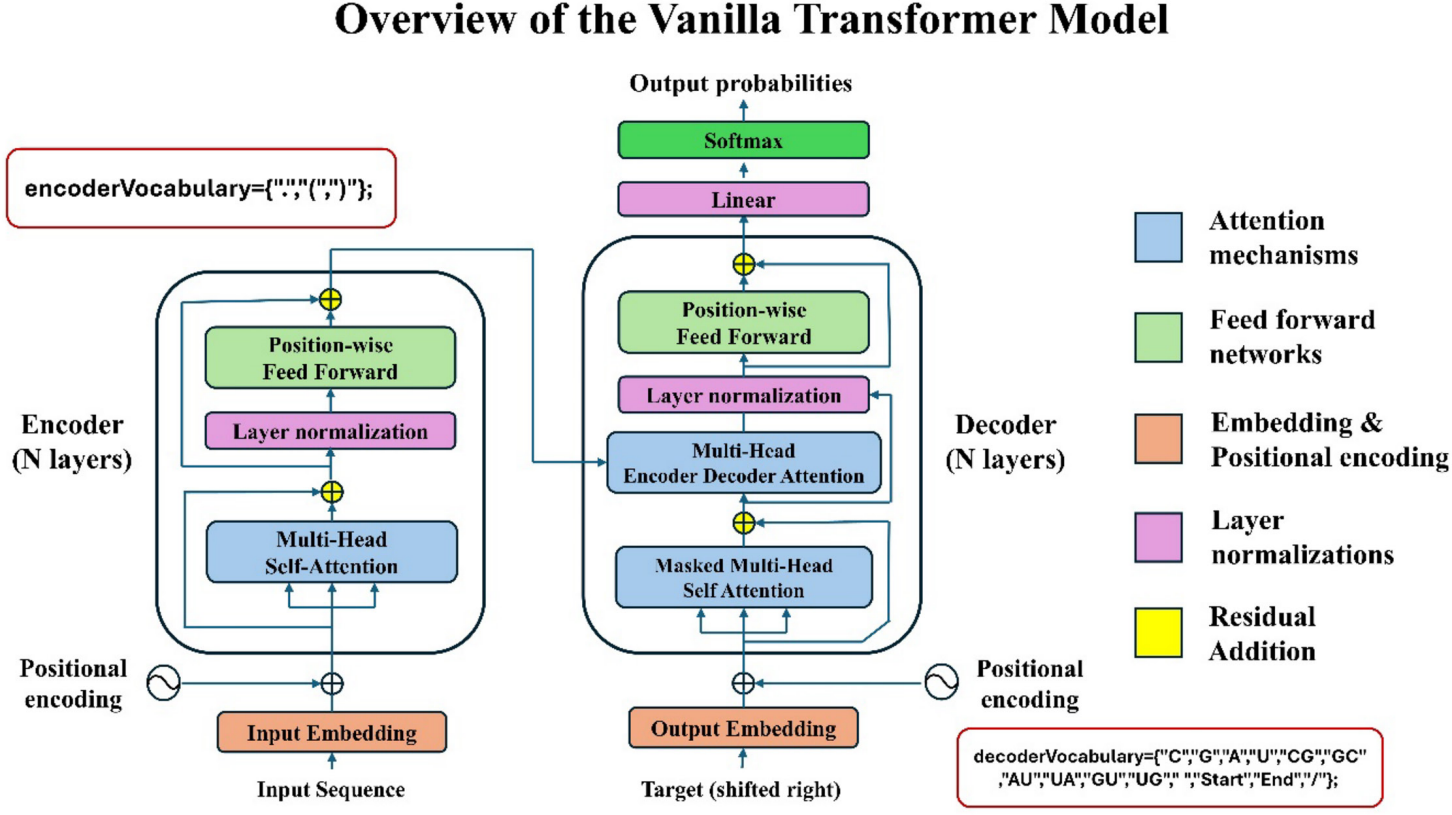
A transformer encoder–decoder neural network before inverse RNA folding. For RNA sequence and secondary structure, the encoder vocabulary consists of dots and brackets, and the decoder vocabulary consists of RNA nucleotides and base‐pairs.

It is worth noting that, although not directly for riboswitch discovery, ML has been applied to other riboswitch‐related research applications (Groher et al. 2019; Kelvin et al. 2026; Fernandez‐de‐Cossio‐Diaz et al. 2025) and general tools such as Svhip (Klapproth et al. 2023) that are related. However, its application to RNA secondary structure prediction from sequence remains limited by the availability and quality of training data (Flamm et al. 2022; Schneider et al. 2023).

## 4 Why ML to Enhance Biophysical Models?

At the core of RNA design lies the direct problem of predicting RNA secondary structure from sequence. Due to limitations in the available training data (Flamm et al. 2022), it remains common practice to rely on biophysical models for this task. Although ML approaches have been explored, for example, RNAformer (Franke et al. 2024), none have yet demonstrated sufficient performance to replace thermodynamics‐based models. For instance, the eteRNA game challenges rely on ViennaRNA folding predictions as ground truth, and top players develop strong intuition for RNAfold predictions. Importantly, this limitation should be understood specifically in the context of the secondary‐structure prediction and inverse‐folding problem considered here. In 3D fixed‐backbone RNA design, recent geometric deep‐learning approaches such as gRNAde (Joshi and Liò 2025) and NA‐MPNN (Kubaney et al. 2025) have achieved strong performance, with gRNAde, for example, outperforming Rosetta in native‐sequence recovery on a fixed‐backbone RNA redesign benchmark. These methods, however, address a distinct problem, as sequence generation is conditioned on a predefined three‐dimensional RNA backbone. In the riboswitch‐discovery setting considered here, the input is a target secondary structure, and an experimentally determined 3D backbone would generally not be available for a potentially undiscovered riboswitch candidate. Thus, although 3D backbone‐conditioned deep‐learning methods represent an important advance in RNA design, they are not directly applicable to the secondary‐structure‐based search framework considered here.

As for riboswitch aptamers, the purine riboswitch is well predicted by energy minimization (Barash and Gabdank 2010), as is the smaller PreQ1 riboswitch (Drory‐Retwitzer et al. 2015). Other riboswitch aptamers, especially larger ones, require sequence constraints during energy minimization prediction. However, in principle, they could still be amenable to energy minimization predictions by thermodynamic models. Therefore, introducing a ML preprocessing step before RNA design, while still relying on thermodynamic models, may provide a promising approach for the discovery of riboswitch aptamers such as SAM, lysine, FMN, glycine, cobalamin, and TPP riboswitches. The latter has already been identified in eukaryotes, specifically in fungi and plants, but it may still serve as a useful system for validation and potentially for discovering additional ones.

To summarize, ML preprocessing is proposed as a strategy to improve initialization for inverse RNA folding by reducing random restarts and unsuccessful Monte Carlo trajectories. A related benefit has recently been demonstrated in a different RNA design context, namely the solution of difficult Eterna game challenges (Cazenave and Touzani 2025). However, for riboswitch identification, the extent of this improvement remains a hypothesis that requires benchmarking. We expect that ML‐based initialization should improve search efficiency, although the magnitude of the benefit must be established empirically. Inverse RNA folding of larger aptamers is expected to become increasingly computationally demanding because the method repeatedly evaluates a forward folding model during Monte Carlo optimization, while the sequence space and structural complexity grow substantially with RNA length. Consequently, glycine, SAM, FMN, lysine, or cobalamin aptamer targets could plausibly require substantially longer runtimes when many restarts and sequence windows are considered. This remains an algorithmic expectation, however, and practical runtime should be measured after careful implementation and optimization of the extensions across different aptamer classes. In this framework, ML preprocessing is intended primarily to improve computational efficiency and initialization quality rather than replacing physics‐based folding models. The evaluation will continue to rely on energy‐minimization folding predictors, ideally incorporating biologically informed constraints derived from known bacterial aptamers, including conserved helices, ligand‐binding residues, covariation‐supported base pairs, and essential sequence motifs. The ML model would therefore provide improved starting sequences, enabling inverse folding algorithms to explore candidate solutions more efficiently with fewer random initializations and unsuccessful optimization trajectories.

A suitable benchmark would compare standard inverse folding against ML‐initialized inverse folding across a range of aptamer lengths and structural complexities, evaluating metrics such as runtime, success rate, number of forward folding evaluations, and final distance from the target structure.

## 5 Conclusion and Future Prospects

At a time when numerous riboswitches have already been identified in bacteria, alongside ongoing efforts to discover additional ones (Narunsky et al. 2024), a variety of strategies are also being developed to identify novel riboswitches in eukaryotes. Most approaches rely on comparative analyses with bacterial riboswitches. Some approaches focus on expected genomic contexts, where prediction confidence is higher (Weinberg, Lünse, et al. 2017; Weinberg, Nelson, et al. 2017; Kavita et al. 2025), whereas others explore less conventional transcriptomic contexts, including searches for RNA structural switches in humans (Khoroshkin et al. 2024; Raymond et al. 2025). Between these extremes are more general search strategies that combine RNA design principles with the state‐of‐the‐art Infernal (Nawrocki and Eddy 2013) for finding riboswitches, as demonstrated by Mukherjee et al. (2023). While Raymond et al. (2025) presents a purely computational machine‐learning‐based approach, other studies combine computational and experimental methods and include RNA structure prediction to varying extents (Mukherjee et al. 2023; Khoroshkin et al. 2024), but do not yet incorporate ML. Despite their methodological differences, such approaches could plausibly benefit from future integration of machine‐learning techniques.

Here, we argue that thermodynamic models for RNA structure prediction do not need to be replaced by ML, particularly given known limitations and biases in the training data (Flamm et al. 2022). Instead, these models can be enhanced through the integration of ML while retaining the advantages of biophysical approaches to RNA secondary structure prediction from sequence. We present one such example within our own research direction (Mukherjee et al. 2023), proposing a strategy that we have begun to develop in which ML is incorporated as a preprocessing step prior to RNA design, as outlined in Figures 1 and 2. Nevertheless, other strategies for integrating ML with biophysical models can also be envisioned. We anticipate that combining novel computational strategies, including ML, with advanced experimental validation methods will lead to new discoveries in riboswitch research.

## Author Contributions

**Rami Zakh:** investigation, writing – original draft, visualization. **Alexander Churkin:** investigation, writing – original draft, visualization. **Ahmed Elbanna:** investigation, writing – original draft, visualization. **Sumit Mukherjee:** conceptualization, writing – original draft, writing – review and editing, investigation, validation. **Danny Barash:** conceptualization, supervision, writing – original draft, writting – review and editing, investigation, validation.

## Conflicts of Interest

The authors declare no conflicts of interest.

## Data Availability

Data sharing not applicable to this article as no datasets were generated or analyzed during the current study.
